# Comparison of the Visibility of Fetal Tooth Buds on 1.5 and 3 Tesla MRI

**DOI:** 10.3390/jcm9113424

**Published:** 2020-10-26

**Authors:** Burkhard Kunzendorf, Mariana C. Diogo, Delfina I. Covini, Michael Weber, Gerlinde M. Gruber, Hans-Florian Zeilhofer, Britt-Isabelle Berg, Daniela Prayer

**Affiliations:** 1 Hightech-Research-Center of Oral and Cranio-Maxillofacial Surgery, Department of Biomedical Engineering, University of Basel, Gewerbestrasse 14, 4123 Allschwil, Switzerland; burkhard-kunzendorf@gmx.de (B.K.); hans-florian.zeilhofer@usb.ch (H.-F.Z.); 2 Department of Radiology, Division of Neuro- and Musculoskeletal Radiology, Medical University of Vienna, Währinger Gürtel 18–20, 1090 Vienna, Austria; mariana.cardoso.diogo@gmail.com (M.C.D.); delfina.covini@hospitalitaliano.org.ar (D.I.C.); michael.weber@meduniwien.ac.at (M.W.); Gerlinde.Gruber@kl.ac.at (G.M.G.); 3 Department of Cranio-Maxillofacial Surgery, University Hospital Basel, Spitalstrasse 21, 4031 Basel, Switzerland; daniela.prayer@meduniwien.ac.at

**Keywords:** dental imaging, fetal MRI, head and neck imaging, magnetic resonance imaging, prenatal diagnosis, tooth buds

## Abstract

Dental anomalies coincide with genetic disorders, and prenatal identification may contribute to a more accurate diagnosis. The aim of this study was to assess whether fetal Magnet Resonance Imaging (MRI) is suitable to visualize and investigate intrauterine dental development in the upper jaw, and to compare the quality of visibility of tooth buds between 1.5 Tesla (T) and 3T images. MR images of fetuses Gestational Week (GW) 26.71 ± 4.97 from 286 pregnant women with diagnoses unrelated to dental anomalies were assessed by three raters. We compared the visibility between groups and field strengths in five gestational age groups, using chi square and Fisher’s exact tests. All ten primary tooth buds were identifiable in 5.4% at GW 18–21, in 75.5% at GW 26–29, and in 90.6% at GW 34+. Before GW 30, more tooth buds were identifiable on 3T images than on 1.5T images. Statistical significance was only reached for identification of incisors (*p* = 0.047). Therefore, 1.5T and 3T images are viable to visualize tooth buds, particularly after GW 25, and their analysis may serve as diagnostic criterion. MRI tooth bud data might have an impact on various fields of research, such as the maldevelopment of teeth and their causes. Analyzing tooth buds as an additional diagnostic criterion is not time consuming, and could lead to an improvement of syndrome diagnosis.

## 1. Introduction

Dental development starts in the fifth gestational week (GW). Between GW 9 and 14, the primary frontal teeth, and between GW 13 and 19, the primary molar teeth, reach the final bell stage. The tooth germ primarily consists of the enamel organ, the dental papilla, and the dental sac [1]. In the course of pregnancy, these structures progressively differentiate, mineralize, and increase in size. This complicated mechanism, however, can be dysfunctional, which may result in alterations of structure, shape, and number of teeth [1]. In the general population, the prevalence of hypodontia is around 1% for primary teeth [2,3], and 6.4% for permanent teeth; these numbers vary among different ethnicities [4].

Many genetic disorders present with an increased prevalence of dental anomalies. A total of 126 known genetic syndromes have oligodontia or anodontia as a clinical sign; the syndromes most frequently associated with oligodontia or anodontia are ectodermal dysplasias, Down’s syndrome, and facial clefts of all types [5]. Furthermore, over 80 genetic syndromes include hypodontia. Sixty to eighty percent of patients with the most common form of ectodermal dysplasia (x-linked hypohidrotic ectodermal dysplasia) carry some form of hypodontia affecting their primary and secondary teeth [6]. A study by Haque and Alam [7] revealed that 66.7% of cleft patients have missing teeth. Furthermore, agenesis of the permanent teeth was observed in 54.6% of patients with Down’s syndrome [8], and a general relationship between low birth weight, high risk pregnancy, and an abnormal number of teeth was reported [9]. Many other causes potentially impair the tooth development, such as medications, toxins (e.g., lead), and infections [10,11]. Little is known about these disruptive mechanisms; therefore, and in order to better understand these processes, visualization of the perigestational dental development is important. In utero, ultrasound (US) imaging can be used [5], but it has its limitations.

Fetal Magnet Resonance Imaging (MRI) is increasingly used as a compliment to ultrasound in prenatal diagnosis [12], in order to improve diagnostic accuracy [13], and also patient counseling and follow up. However, the literature on the evaluation of tooth buds in fetal MRI is limited to a few case reports [14,15]. No systematic study has been published to date that analyzes the visibility of fetal tooth buds throughout pregnancy using fetal MRI. Studies using ultrasound, however, could prove the feasibility and usefulness of displaying fetal tooth buds [16].

Therefore, our goal was to assess whether fetal MRI is suitable to visualize and investigate intrauterine dental development in the upper jaw in a healthy collective, and to compare the quality of visibility of tooth buds between 1.5T and 3T images.

## 2. Materials and Methods

### 2.1. Patients and Setting

The cases were retrospectively collected from a database of clinically indicated fetal MR examinations of the neuroradiology department of a single tertiary center. The study was approved by the local ethics committee (Ethical committee Medical University Vienna, Vienna, Austria, 1329/2016). Informed consent was obtained prior to imaging.

**Figure 1:**
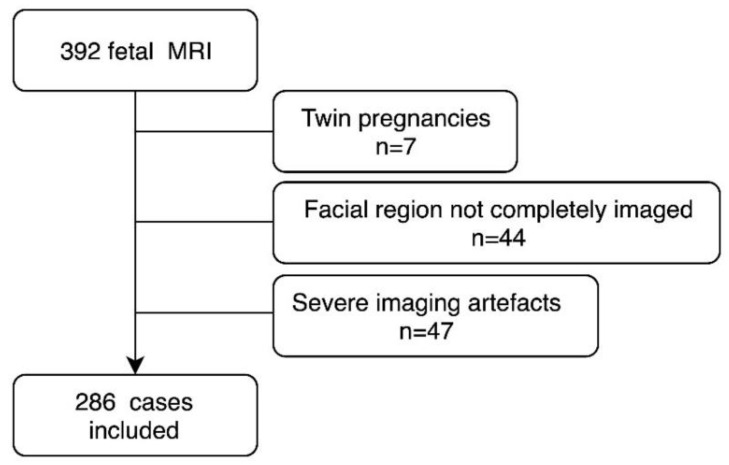
Diagram representing exclusion criteria applied in the current study.

We included cases acquired between 1 January 2009 and 31 December 2015, with a known ultrasound-confirmed gestational age, who had had axial T2-weighted single shot fast spin echo (ssFSE) images over the fetal head, and who had no known or detected dental anomalies or facial skeletal abnormalities and chromosomal anomalies. A diagram of further exclusion criteria can be found in Figure 1. The medical diagnosis, based on the fetal MRI, was classified into: no abnormalities, brain abnormalities, thoracic abnormalities, abdominal abnormalities, limb abnormalities, and extra-fetal abnormalities (Table 1).

### 2.2. Imaging Analysis

Examinations were performed for different medical fetal and maternal indications and were not acquired specifically to image the fetal maxilla. Images were acquired on both a 1.5T MR system (Philips Ingenia, Best, The Netherlands) with a six-element body phase-array coil and a 3T MR system (Philips Achieva, Best, The Netherlands) with a five-element cardiac surface coil. The women were scanned in the supine or left lateral decubitus position; neither fetal nor maternal sedation was used.

The sequence parameters of the T2-weighted ssFSE images were: on 1.5T: 200–230 mm field of view (FOV), 256 × 153 matrix, TR highest, TE 140 ms, FA 90, slice thickness 3–5 mm with 0–0.5 mm gap; and on 3T: 250 FOV, 228 × 204 matrix, TR highest, TE 200 ms, and a 3 mm slice thickness with 0.3 mm gap.

**Figure 2:**
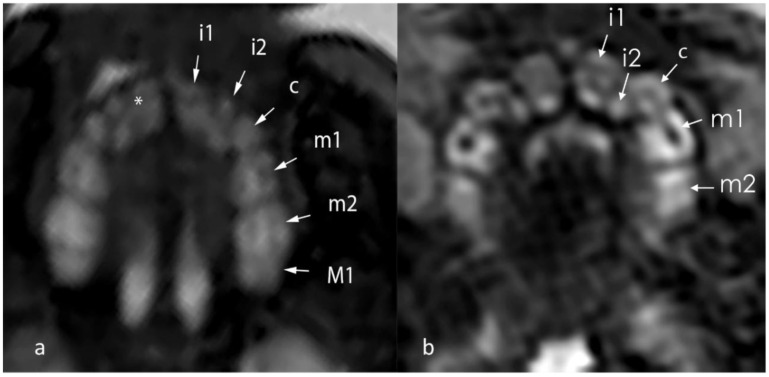
T2-weighted ssFSE axial images of two GW 36 fetus, performed at 1.5T (**a**) and 3T (**b**) displaying the tooth buds: i1 (first primary incisivus), i2 (second primary incisivus), c (primary caninus), m1 (first primary molar), m2 (second primary molar), M1 (first permanent molar). The asterisk shows the calcified edge within the tooth bud.

MR examinations were independently reviewed by a neuroradiologist (M.C.D.) and a radiologist (D.C.) with over five years of experience and special training in fetal MRI, as well as by a maxillofacial surgeon (B.K.) with two years of experience in fetal MRI, blinded to all clinical information. Each rater assessed whether or not the identification of the dental lamina was possible, and categorised it in 5 grades: “1” no tooth buds visible, “2” 1–5 tooth buds visible, “3” 6–8 tooth buds visible, “4” nine tooth buds visible, “5” ten tooth buds visible (Figure 2).

The raters were further asked to mark the specific location of the visualized tooth buds on a schematic drawing of the upper jaw, based on expected tooth bud development (16).

### 2.3. Statistic Analysis

The cases were clustered into five age groups (GW 18–21, GW 22–25, GW 26–29, GW 30–33, and GW 34+). Tooth buds were considered visible if they were identified by at least two raters. The tooth buds of the primary teeth were analyzed. Crosstabs and Fisher exact tests were used to compare the visibility of tooth buds between 1.5T and 3T, separately for each age group. A chi^2^ test was used to compare the distribution of the age groups of all cases examined with the 1.5T and 3T machines. All tests were performed at a significance level of α = 0.05. All statistical computations were performed using IBM SPSS (version 23.0 for Windows, IBM Cor, Armonk, NY, USA).

## 3. Results

A total of 286 fetal MRI examinations were included, with a mean (SD) age of GW 26.71 (4.97), ranging from GW 17 + 0 to GW 37 + 3. There was no significant difference in age distribution between 1.5T and 3T cases, neither when considering each GW separately (*p* = 0.282) nor as groups (GW 18–21, GW 22–25, GW 26–29, GW 30–33, and GW 34+; *p* = 0.177). A detailed age distribution and the medical diagnosis after MR imaging are depicted in Table 1. In total, 170 data sets were acquired on a 1.5T MR system and 116 data sets were acquired on a 3T MR.

**Figure 3:**
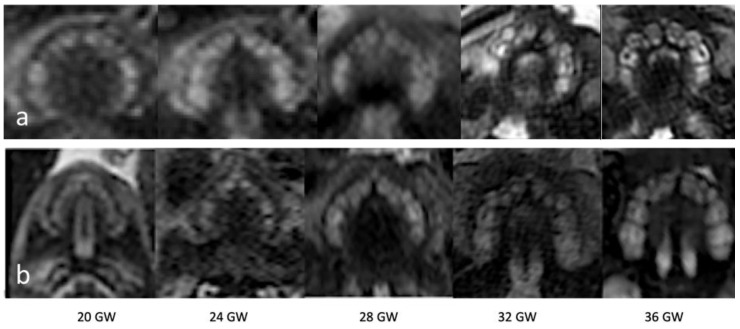
T2-weighted ssFSE images performed with a 3T MRI (**a**) and with an 1.5T MRI (**b**) of the dental lamina from GW 20–36, showing a gradual increase in tooth bud visibility, with all tooth buds identifiable at GW 36.

Tooth buds appear as hyperintense round structures within the dental lamina on T2-weighted sequences, with the calcified part of the tooth appearing as a hypointense line within the buds (Figure 2). With advancing gestational age, the tooth buds increase in size and can more easily be identified as distinguished, separate structures, aligned within the maxilla (Table 2, Figure 3).

**Figure 4:**
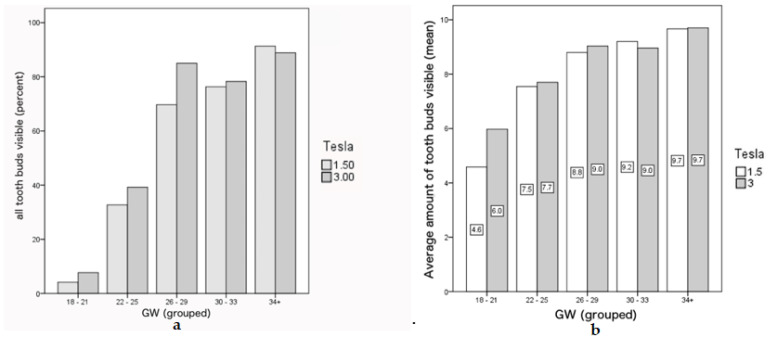
Comparison of 1.5T and 3T images with regard to the GW groups and visibility of tooth buds. (**a**) Percentage of cases per age group where all tooth buds were visible in both the 1.5T and 3T Images. (**b**) Average amount of tooth buds visible in different age groups, again in both the 1.5T and 3T Images.

At GW 18–21, only 5.4% (2/37) of the cases had all tooth buds identifiable, with an average of 4.6 and 6.0 tooth buds identified on 1.5T and 3T scans, respectively. This number increases with gestational age, reaching 90.6% (29/32) at GW 34–38, with an equal average of tooth buds identified in both field strength (9.7). The number of identifiable tooth buds per field strength and bud visibility scoring per gestational age group are presented in Table 2. In earlier gestations, a higher number of tooth buds was identifiable on the 3T MR images compared to 1.5T images (Table 2), although this difference did not reach statistical significance (*p* = 0.810) (Figure 4). The incisor tooth buds were overall better visualized on 3T MR images (*p* = 0.047). However, when analyzing each incisor separately, this superiority was only statistically significant for the 1st (*p* = 0.038) and second incisors (*p* = 0.046) on the right side and when analyzing gestational age subgroups, it was only the 1st incisor, and below GW 26 (GW 18–21: *p* = 0.015; GW 22–25: *p* = 0.046).

No significant differences regarding tooth bud visibility were found for canines (*p* = 0.390) and molars (*p* = 0.259; m1: *p* = 1.00; m2: *p* = 0.259) between 1.5T and 3T images.

**Figure 5:**
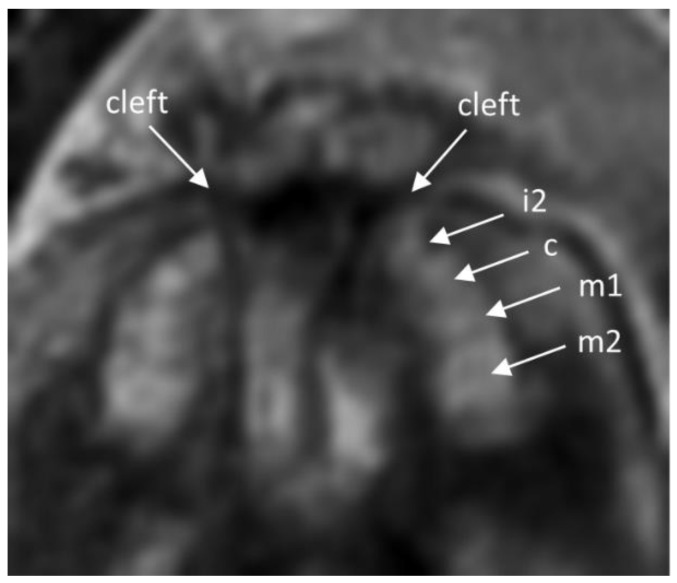
The T2-weighted ssFSE image 1.5T image shows the upper jaw of a patient in GW 27 with a bilateral cleft lip and palate. The frontal tooth buds are not visible. Tooth buds i2 (second incisor), c (canine), m1 (first molar), and m2 (second molar) are visible.

Figure 5 shows an image of a patient in the GW 27 with a bilateral cleft with missing first frontal tooth buds as example for future diagnostic purposes.

**Figure 6:**
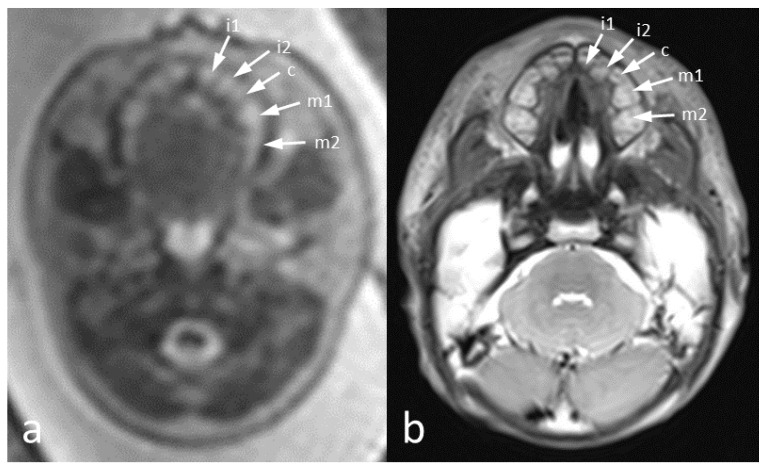
The T2-weighted images above show the dental arch in the GW 24 in vivo (**a**) and a post mortem image (**b**) of the same patient. In both images, all primary tooth buds are visible: i2 (first incisivus), i2 (second incisicus), c (canine), m1 (first molar), and m2 (second molar).

Figure 6 shows a comparison of an intrauterine MRI scan and a post-mortem MRI scan of the same patient. Tooth buds are visible and detectable in both but the post-mortem quality is significantly better.

## 4. Discussion

### 4.1. Principal Findings

Identification of the dental lamina and individual tooth buds of the maxilla is possible using routine, non-directed fetal brain MRI. 

While US is the preferred modality to visualize prenatal tooth buds [17,18,19], it has never reached clinical routine use. Furthermore, fetal US is operator-dependent and has inherent limiting factors such as maternal habitus, oligohydramnios, and the position of the fetal face during the examination [5]. Publications on in utero MRI of the maxillofacial region are readily available, mostly concerning facial clefts, but also fetal lacrimal pathways and eye movement, showing that it is possible to detect small craniofacial structures [20,21], including the maxillary arch [22]. In this study, we further demonstrate that routine fetal MRI is a viable tool to visualize individual fetal tooth buds. On mid-second trimester (GW 18–21) scans, tooth buds were identifiable in 92.8% of fetuses, although all (ten) buds were only visible in 1% of the cases. With advancing gestational age, the tooth buds increase in size and could more easily be identified as distinguished, separate structures, aligned within the maxilla. This increased during gestation, with at least one bud visible in all examinations from GW 22, and with 80% of grade 5 visibility (10 tooth buds) in the older gestational age group (GW 34–38). Molar tooth buds appear more squared and bigger in size when compared to the frontal and canine buds. After GW 24, the frontal tooth buds appeared to be more cramped against each other.

### 4.2. Results

Our study provides important reference data concerning the visibility of tooth buds for the different gestational ages. It further illustrates a potential advantage of using the 3T MR imaging, particularly in earlier gestations (<GW 26). The increase of field strength using a 3T MRI leads to a higher signal-to-noise ratio, resulting in a higher temporal and/or spatial resolution, which allows for an improved visualization of small parts [23].

From our experience, using data sets that were not designed to have the focus on fetal dentition, 3T MR images are superior to 1.5T images for the visualization of fetal tooth buds.

### 4.3. Clinical Implications

Irrespective of the magnetic field strength, description of tooth bud visualization might help future investigators to evaluate prenatal dentition. Identifying tooth buds can have clinical implications, as numerous pathological entities manifest with anomalies of their shape and/or count. Using fetal MRI, adontia may be excluded as early as in GW 22 (or earlier, if buds can be identified). Oligodontia can be excluded whenever 9 teeth buds are visible, and hypo- and hyperdontia may be diagnosed by determining the exact number of tooth buds, probably in later gestational ages. Other entities, such as hypohidrotic ectodermal dysplasias [15], may also be identified, complementing or confirming ultrasound findings. Application of individual tooth bud visualization is also relevant in much more common entities, such as in cleft-lip-palate [18]. The incisor buds were most often identified from an earlier gestational age on fetal MRI. This is of great interest for clinicians, since the upper lateral incisor is the most susceptible to injury in the area of the cleft in both deciduous and permanent dentitions [24] in patients with cleft-lip-palate, and are the permanent tooth with the highest prevalence of agenesis [8] in Down’s syndrome patients. In non-syndromic hypodontia, the maxillary lateral incisor is the second most commonly missing tooth [4].

If the primary teeth are not present or removed, neither primary dentition nor its permanent successor will develop [17,25]. If missing tooth buds are diagnosed on fetal MRI, this will have implications on the dentition of childhood and adulthood.

Research implications:

Aka et al. [26] published a study assessing central incisors of dead fetuses using manual measurements (length/width, crown, root). They concluded that age estimation was accurate within ±0–2 weeks, and that forensics should take the fetus dentition period into account when assessing the embryonic development [26]. Fetal MR imaging could enable such measurements in living fetuses too, which were not performed in the current study.

From our point of view, it is desirable to take tooth buds into account during an MRI scan, if diseases with teeth abnormalities are suspected. Specifications in the fetal MR sequences could improve image quality, but it is not reasonable to perform an MR series only to display tooth buds.

### 4.4. Strength and Limitations

The visibility of tooth buds may improve by directed fetal MRI acquisitions if dental pathologies are suspected, as the results presented originate from images directed at the fetal brain for diverse indications, with axial images oriented perpendicularly to the brainstem and not aimed at optimal dental visualization. Using the hard palate as a reference point would place the diagnostic plane in line with the tooth buds.

The performance of a fetal MRI is generally considered to be safe. No study could demonstrate any definitive risks for the fetus or the mother if the MRI scanners are operated according to the regulatory guidelines, for example by the Food and Drug Administration. Going from 1.5T to 3T in field strength, the static magnetic field exposure, the radiofrequency power deposition, and the specific absorption rate as well as the acoustic noise and sound pressure level change; however, even the 3T field strength has no harmful effect, neither on the mother nor the fetus [23].

Limitations of this study also include those inherent to the retrospective design; furthermore, we had a small sample size with a limited number of cases per gestational age. As in other studies analyzing small fetal head structures [27], we have noticed image artifacts (such as motion artifacts) that may have influenced the analyses. In addition, we have omitted collecting postnatal follow-up data that could have confirmed dentition development; even if we have tried our best to exclude any known causes of abnormal dentition, there may have been some cases in our cohort. The clinical application in specific syndromes also remains to be studied.

## 5. Conclusions

This study is the first systematic analysis on the visibility of tooth buds on fetal MRI, and yields valuable reference data. We could show that both 1.5T and 3T images are viable to visualize tooth buds, particularly after GW 25, and their analysis may serve as diagnostic criterion. MRI tooth buds data might have an impact on various fields of research, such as in the maldevelopment of teeth and their causes. Analyzing tooth buds as an additional diagnostic criterion is not time consuming and could lead to an improvement of syndrome diagnosis.

## Figures and Tables

**Table 1:** Distribution of patient images by gestational week (GW) and main reason for fetal Magnet Resonance Imaging (MRI) referral, by organ system.

GW Group	18–21	22–25	26–29	30–33	34+	Total (*n*)	Total (%)
**1.5 Tesla**							
*n*=	23	51	33	39	24	170	100%
no abnormalities	1	9	0	11	10	31	18%
brain abnormalities	7	16	16	17	7	63	37%
thoracic abnormalities	7	10	7	6	2	32	19%
abdominal abnormalities	7	12	8	3	3	33	19%
extra-fetal and limp abnormalities	1	4	2	2	2	11	7%
**3 Tesla**							
*n*=	13	50	20	23	10	116	100%
no abnormalities	1	6	1	5	1	14	12%
brain abnormalities	7	21	10	12	4	54	46%
thoracic abnormalities	5	13	3	2	1	24	21%
abdominal abnormalities	0	7	4	2	2	15	13%
extra-fetal and limb abnormalities	0	3	2	2	2	9	8%

**Table 2:** Number of cases where all tooth buds were visible, mean (SD) number of tooth buds visible, and allocation to the categories, separately for each gestational age group. Mean (SD) number of visible tooth buds in each age group as well as allocation to the preset categories are shown in Table 2. Grading scale in categories (Cat.): “1” no tooth buds visible, “2” 1–5 tooth buds visible, “3” 6–8 tooth buds visible, “4” nine tooth buds visible, “5” ten tooth buds visible.

GW	All Tooth Buds Visible *n*/Total (%)	1.5T		3T		Cat. 1 (%)	Cat. 2 (%)	Cat. 3 (%)	Cat. 4 (%)	Cat. 5 (%)
		Mean	SD	Mean	SD					
GW 18–21	2/37 (5.4)	4.6	2.9	6.0	1.9	7.2	45.0	37.8	9.0	0.9
GW 22–25	37/103 (35.9)	7.6	2.4	7.7	2.1	0.3	17.6	41.8	14.4	25.8
GW 26–29	40/53 (75.5)	8.8	1.8	9.0	1.8	0.0	7.5	18.9	16.4	57.2
GW 30–33	47/61 (77)	9.2	1.1	9.0	1.6	0.0	1.6	25.7	14.8	57.9
GW 34–38	29/32 (90.6)	9.7	0.7	9.7	0.7	0.0	0.0	8.3	11.5	80.2
